# Supplementary material for: MetaRibo-Seq measures translation in microbiomes
Source: Nat Commun. 2020 Jun 29;11:3268. doi: 10.1038/s41467-020-17081-z (PMC7324362; doi:10.1038/s41467-020-17081-z)
Supplement: Supplementary file 10 — Supplementary Data 7 [file 41467_2020_17081_MOESM10_ESM.zip › File2/Confidence_VeryHigh_Taxonomy/305883_out.krona.html]

Javascript must be enabled to view this page.

members
magnitude
magnitudeUnassigned
count
unassigned
taxon
rank

305883\_out

27

superkingdom
27
2

phylum
27
976

class
27
200643

order
27
171549

815
27
family

genus
27
816


SRS1055083\_contig\_number\_8095
1
species
2292941

291645
1

SRS1041134\_contig\_number\_3572
species

310298

SRS011239\_contig\_number\_26545SRS014459\_contig\_number\_40593SRS014534\_contig\_number\_1929SRS015065\_contig\_number\_37207SRS015663\_contig\_number\_24391SRS015782\_contig\_number\_29980SRS017375\_contig\_number\_101SRS023715\_contig\_number\_contig-100\_2163.177938SRS047044\_contig\_number\_4719SRS047433\_contig\_number\_862SRS051031\_contig\_number\_23687SRS055017\_contig\_number\_29880SRS1041164\_contig\_number\_3553SRS143070\_contig\_number\_10149SRS150029\_contig\_number\_12112SRS971275\_contig\_number\_31823
16
species

species
2

SRS1041143\_contig\_number\_6073SRS147271\_contig\_number\_23999
1262735

species
2

SRS013098\_contig\_number\_33752SRS063518\_contig\_number\_34102
1263040


SRS013382\_contig\_number\_1467
1
species
1262739

1

SRS054352\_contig\_number\_17303
species
1638779


SRS017433\_contig\_number\_10383SRS078665\_contig\_number\_7254SRS143070\_contig\_number\_4983
3
species
310297
